# CD11c-Cre driven deletion of *Irf8* reveals the effect of somatic mosaicism in a mouse model of SLE

**DOI:** 10.3389/fimmu.2025.1662894

**Published:** 2026-01-26

**Authors:** Hongsheng Wang, Chen-Feng Qi, Bethany Scott, Hemanta Kole, Silvia Bolland

**Affiliations:** Laboratory of Immunogenetics, National Institute of Allergy and Infectious Diseases, National Institutes of Health, Rockville, MD, United States

**Keywords:** DCs, FcγRIIB, glomerulonephritis, lupus, transcription factors

## Abstract

The pathogenesis of systemic lupus erythematosus (SLE) is caused by a complex mix of genetic factors that lead to dysregulation of the immune response. Mild susceptibility or resistance factors can tilt the scale towards or against pathology. Here, we present evidence for the *Irf8* gene as a lupus protective factor in conditions of haploinsufficiency or mosaicism. We targeted *Irf8* expression in mice deficient in *Fcgr2b*, a well characterized mouse model of SLE. As is the case in human SLE, hyperresponsive B cells and dendritic cells (DCs) are causal factors at various stages of disease in *Fcgr2b*-deficient mice (*R2^-/-^*). Since *Irf8* is essential for the generation of cDC1s, we used conditional deletion with various known DC-targeting Cre systems to delete *Irf8*. All conditional systems tested to delete *Irf8* reduced the titer of antinuclear antibodies and abrogated kidney pathology in *R2^-/-^* mice. In addition to the expected effect of *Irf8* deletion in cDC1s, we unexpectedly found that mosaic deletion of *Irf8* also occurred in B cells and other immune cells. Using mixed bone marrow chimeras we determined that the aborted disease in *Irf8^f/f^CD11c-Cre+R2^-/-^* and *Irf8^f/f^Itgax-Cre+R2^-/-^* mice could be attributed to the inability of B cells with partial reduction of IRF8 to produce autoantibodies. Therefore, these results reveal IRF8 as a susceptibility factor of SLE even in cases of mild changes of expression levels and mosaic somatic deletion of the gene in B cells.

## Introduction

Systemic lupus erythematosus (SLE) is a chronic disease that affects multiple organ systems. Kidney involvement often has severe consequences, such as organ failure. Elevated production of autoantibodies against nuclear antigens is the hallmark of SLE. Immune complex deposition in the kidney is thought to initiate the inflammatory response of SLE by attracting many immune cells to produce inflammatory factors, which eventually induce irreversible tissue damage, proteinuria, and kidney failure. Several murine models have been investigated to uncover the mechanisms of the pathogenesis of SLE (1–3). In one of these models, mice bearing an *Fcgr2b* null allele (designated *R2^-/-^*) spontaneously develop lupus-like symptoms, including elevated levels of anti-nuclear antibodies (ANA), proteinuria and glomerulonephritis in a B cell-dependent manner (4). It is unclear whether other cell types, such as conventional dendritic cells (cDCs), also play a role. cDCs of both mice and humans contain two phenotypically and functionally distinct subsets, cDC1s (XCR1^+^) and cDC2s (CD172α^+^) (5). cDC1s perform antigen cross-presentation to CD8^+^ T cells, whereas cDC2s present exogenous antigens to CD4^+^ T cells (5). The functions of cDC1s in the pathogenesis of arthritis and biliary cholangitis have been reported previously (6, 7). However, the role of cDC1s in SLE and glomerulonephritis remains poorly understood.

The lineage specification and commitment of cDC1s and cDC2s are regulated by a group of transcription factors (8). IRF8 is one of the master transcription factors required for the development of cDC1s (9–13). Given its broad expression in immune and non-immune cells, IRF8 regulates gene programs involved in a variety of cellular functions, such as differentiation, interferon signaling, metabolism, and survival (14–16). Mutations in *Irf8* in humans have been associated with infectious disease (17). GWAS analyses have revealed strong association of IRF8 polymorphism with increased susceptibility to SLE (18). Previous studies by our group and others have identified IRF8 as an important regulator of B cell development and function (16, 19, 20). While conditional deletion of IRF8 using CD19-Cre resulted in slightly expanded marginal zone B cells under physiological conditions (21), a deletion of IRF8 using CD23-Cre abrogated lupus symptoms in DEF6 and SWAP-70 double knockout mice, which has been used to model SLE (22). While a homozygous mutation of IRF8 often induces more severe symptoms in humans, such as immunodeficiency and infectious diseases (23), heterozygous mutation of IRF8 can also cause atypical infectious symptoms (17). These observations suggest that IRF8 may function differently under varying protein concentrations. In fact, a dose-dependent effect of IRF8 has been documented previously in the differentiation of cDC1 (10, 24), DC3 (25), monocytes (26), and NK cells (27). However, these studies were performed using healthy mice. Whether this dose effect of IRF8 also exists under pathological conditions remains to be determined.

To better understand the role of cDCs in the pathogenesis of lupus, we generated IRF8 conditional deletion mice using a floxed IRF8 (*Irf8^f/f^*) and a variety of DC-targeting Cre CD11c- systems under the lupus-prone *R2^-/-^* genetic background. We combined the *Irf8^f/f^* allele in lupus background with CD11c-Cre (28) and Itgax-Cre-EGFP (29), two strains that express Cre under the *Itgax* (also known as *CD11c*) gene promoter but using distinct transgenic constructs. Itgax-Cre-EGFP allows to quantify Cre expression as equimolar to EGFP protein expression. We also tested conditional deletion of *Irf8* using Xcr1-Cre, which expresses Cre specifically in cDC1 cells (30). Conditional deletion of *Irf8* with both types of CD11c-targeted Cre showed profound reduction of lupus phenotypes in *R2^-/-^* mice. However, we discovered that conditional deletion of *Irf8* floxed alleles was leaky in all three systems. The Xcr1-Cre crossbreeding resulted in total deletion of *Irf8* gene. The two strains with Cre targeted to the *Itgax* (CD11c) gene also induced mosaic deletion of *Irf8* in many non-CD11c-expressing immune cells. Our experiments uncover a weakness in CD11c-Cre systems but also provide strong evidence that the gene dose effect of *Irf8* profoundly affects B cell biology.

## Materials and methods

### Mice

*R2^-^*^/-^ mice were obtained from the Taconic National Institute of Allergy and Infectious Diseases colony. IRF8^f/f^ mice have been described previously (21). XCR1-Cre (JAX#035435), CD11c-Cre (JAX#008068) and Itgax-Cre-EGFP (JAX#007567) mice were purchased from Jackson Laboratory. The construct to generate CD11c-Cre mice includes the entire *Cd11c* gene from BAC clone RP24-361C4 and replaces the first exon for Cre recombinase (28). In the construct to generate Itgax-Cre-EGFP mice, 5.3kb of the *Itgax* (*CD11c*) promoter/enhancer directs bicistronic Cre and EGFP protein expression (29). Genotyping and gene excision analysis were performed by Transnetyx. All mice were maintained under specific pathogen-free conditions. Animal studies were conducted according to a protocol approved by the National Institute of Allergy and Infectious Diseases Animal Care and Use Committee.

### Flow cytometry and antibodies

Spleen, lymph nodes, and bone marrow (BM) single cell suspensions were prepared and stained with antibodies listed in **Table 1**. Cells were analyzed with a FACSymphony flow cytometer (BD Biosciences) and FlowJo software (Treestar, Version 10.8.1).

**Table 1:** List of antibodies used in FACS and ANA assays.

Antibody	Source	Application	Clone	Format
CD3	BD	FACS	145-2C11	BB700, PE, PE-CF594
CD24	Biolegend	FACS	M1/69	BV421
CD23	BD	FACS	B3B4	PE.Cy7, BV421
Ly6G	BD	FACS	1A8	PE-CF594
B220	Biolegend	FACS	RA3-6B2	APC.Cy7, Alexa Fluor 700
IgM	Biolegend	FACS	RMM-1	PE.Cy7, APC, FITC
IgD	Biolegend	FACS	11-26c.2a	FITC, BV480
CD95	BD	FACS	DX2	PE, PE.Cy7
CD138	BD	FACS	281-2	PE, APC
CD19	Biolegend	FACS	6D5	APC.Cy7, Alexa Fluor 700, BV785, BV480
CD11b	Biolegend	FACS	M1/70	BV605, BV711, PE.Cy7
CD11c	Biolegend	FACS	N418	BV711, PE.Cy7
CD8	eBioscience	FACS	53-6.7	BV605, BV711, BUV395
CD45	BD	FACS	30-F11	BV421, BUV737
GL7	BD	FACS	GL7	APC
CD4	Biolegend	FACS	GK1.5	APC.Cy7, BUV395
IgM^a^	Biolegend	FACS	MA-69	PE.Cy7
IgM^b^	Biolegend	FACS	AF6-78	PE
IRF8	ThermoFisher	FACS	V3GYWCH	PerCP-eFluor 710
CD45.2	Biolegend	FACS	104	PE-CF594
IgG2a/c[a]	BD	ANA	8.3	Biotin
IgG2a/c	BD	ANA	5.7	Biotin
NK1.1	Biolegend	FACS	S17016D	Alexa Fluor 700, APC.Cy7
F4/80	Biolegend	FACS	BM8	APC, BUV395
I-A/I-E	Biolegend	FACS	M5/114.15.2	APC, BV480
Ly6C	BD	FACS	HK1.4.rMAb	BB700
Mouse IgG (minimal x-reactivity)	Biolegend	FC, IHC-F	Poly4053	Alexa Fluor 488
IgD	BD	FACS	11-26c.2a	BV480
TCRb	Biolegend	FACS	H57-597	PE, Alexa700, BV711
CD26	Miltenyi Biotec	FACS	REA1196	PE
CD21/35	BD	FACS	7G6	PE
AA4.1	Biolegend	FACS	AA4.1	PE.Cy7

For preparing kidney leukocytes, a detailed protocol has been reported recently (31). Briefly, mice were injected with anti-CD45-BV421 antibodies (3 μg in 200 μl) i.v. for 3 min and euthanized immediately. Kidneys were minced with a scissor and digested with the Multi Tissue Dissociation Kit 1 (Miltenyi Biotec) reagents on a GentleMACS Octo Dissociator (Miltenyi Biotec), followed by enrichment for CD45^+^ leukocytes using anti-CD45 microbeads (clone 30F11.1, Miltenyi Biotec) and AutoMACS Pro Separator sorting (Miltenyi Biotec). The eluted cells were then stained and analyzed by flow cytometry.

### BM chimera

Approximately 1x10^7^ of mixed BM cells of *Irf8^f/f^Itgax-Cre-* (Igh^b^), *Irf8^f/f^Itgax-Cre+* (Igh^b^), and R2^-/-^ (Igh^a^) mice at a ratio of 1:1 was injected intravenously into lethally irradiated R2^-/-^ (Igh^b^) mice that received a dose of 940 rad 1 day earlier. Three months later, the reconstituted mice were analyzed for autoantibody production and cellular distribution by flow cytometry.

### Proteinuria and ANA titer testing

Urinal protein levels were measured with Chemstrip 2GP urine test strips (Roche) according to the manufacturer’s instruction. A Chemstrip was dip into freshly voided urine specimen. A color change from yellow to light green/green occurred within 2 min. Results were obtained by direct visual comparison with the color scale printed on the vial label. Proteinuria was continuously monitored from once daily to once weekly. Protein concentration scores of 0, +1, +2, +3 and +4 correspond to a protein concentration of 0, <30, 30, 100 and 500 mg/dL, respectively. Serum ANA titers were determined by the Hep-2 system described previously (32). Briefly, serum samples were diluted at 1:100, 1:300, 1:900 and 1:2700 with PBS and incubated with Hep-2 substrate slides (MBL, AN-1012) at room temperature for 30 minutes. After washing the slides twice with PBS for 5 minutes, the slides were incubated with a secondary anti-mouse IgG-Alexa488 antibody (minimal x-reactivity) (Biolegend) in the dark at room temperature for 30 minutes. In some experiments, secondary antibody was biotinylated anti-mouse IgG2a/c[a] or IgG2a/c[b] (**Table 1**), which was revealed by streptavidin-FITC. Afterwards, the slides were washed with PBS three times. Then, they were imaged under a fluorescence microscope and images were taken. ANA titers were scored as follows: 0= negative at 1:100 dilution; 1= positive at 1:100; 2= positive at 1:300; 3= positive at 1:900; 4= positive at 1:2700; 5= positive at 1:9100 dilution.

### Histology

Kidney tissues were fixed and sectioned by American Histolabs (Gaithersburg, Maryland) for H&E staining. The glomerulonephritis scoring was done by measuring several pathological entities as reported previously (33). The slides were read by a pathologist independently and blindly. Images were taken with an Olympus BX41 microscope (10x and 40x objectives) equipped with an Olympus DP71 camera.

### Statistics

Data were analyzed and figures were made using GraphPad Prism (version 9.0.2). For pairwise comparisons, the appropriate parametric (unpaired Student’s *t*-test) or non-parametric (Mann-Whitney test) was performed. For multiple Mann-Whitney tests on the same set of data, the Bonferroni’s correction tests were carried out.

## Results

### *Irf8* deletion by Itgax-Cre or CD11c-Cre abolishes lupus symptoms in R2^-/-^ mice

R2^-/-^ mice spontaneously develop ANA and proteinuria starting from 3 months of age, which leads to premature death after 5 months of age (4). Hyperresponsive B cells of R2^-/-^ mice are responsible for production of autoantibodies and the initiation of lupus-like symptoms. We observed that the number of inflammatory DCs in the kidney positively correlated with the severity of glomerulonephritis (34), raising the possibility that cDCs may contribute to the pathogenesis of autoimmune nephritis. To gain deeper insight of cDC subsets in regulation of autoimmunity, we used CD11c-Cre, a broadly employed transgenic model to target genes in DCs, to delete floxed *Irf8*, thereby depleting cDC1s (10). As an alternative approach, we used Itgax-Cre-EGFP to delete *Irf8* by taking advantage of EGFP tracking of deleted cells. The *Itgax* gene encodes for CD11c and we therefore expect the same result as in CD11c-Cre lines. By crossing *Irf8^f/f^* with these two Cre lines under the R2^-/-^ background, we generated IRF8-deficient (designated *Irf8^f/f^CD11c-Cre+R2^-/-^* and *Irf8^f/f^Itgax-Cre+R2^-/-^*, respectively) and -sufficient (designated *Irf8^f/f^CD11c-Cre-R2^-/-^* and *Irf8^f/f^Itgax-Cre-R2^-/-^*, respectively) mice. Littermate mice were used throughout this study.

**Figure 1:**
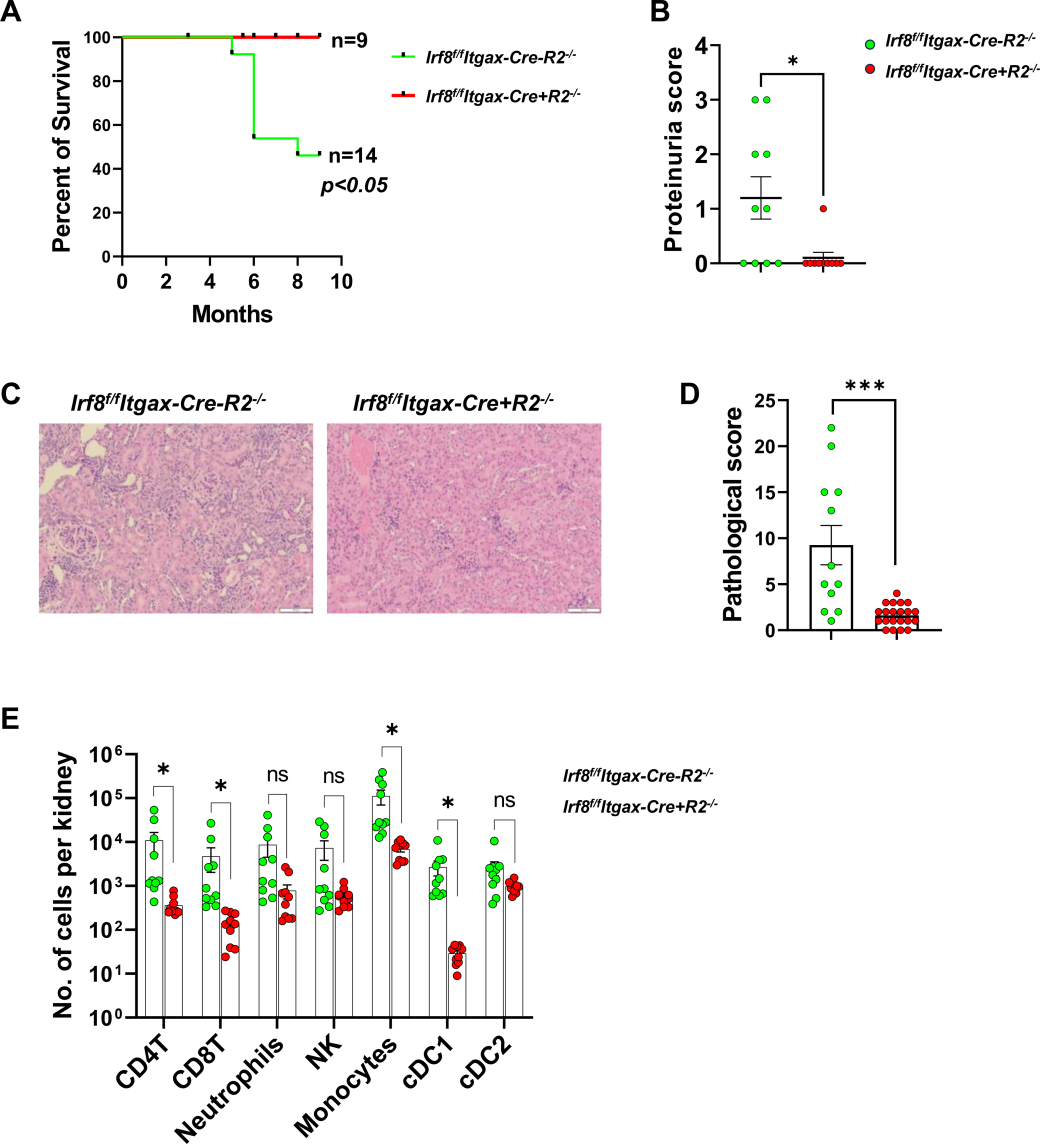
Autoimmunity is abrogated by IRF8 deficiency. **(A)** Survival curves of *Irf8^f/f^Itgax-Cre+R2^-/-^* mice compared to controls. **(B)** proteinuria scores were compared between *Irf8^f/f^Itgax-Cre+R2^-/-^* and control mice. **(C)** Representative H&E-stained sections of kidneys in *Irf8^f/f^Itgax-Cre-R2^-/-^* mice and *Irf8^f/f^Itgax-Cre+R2^-/-^* mice. **(D)** NIH activity index values of **(C)** for each kidney samples studied. **(E)** Numbers of infiltrated subpopulations of cells in each kidney were detected by flow cytometry. **(B, D, E)** Color coding: green for *Irf8^f/f^Itgax-Cre-R2^-/-^* and red for *Irf8^f/f^Itgax-Cre+R2^-/-^* will be carried out for all figures in the manuscript. Each symbol represents a mouse. Statistical significance was calculated in A using comparison of survival curves, **(B, D)** using an unpaired *t* test, and **(E)** using multiple Mann-Whitney tests with Bonferroni’s correction. For **(A, B, D)**, *p < 0.05, ***p < 0.001; for **(E)**, * denotes pairs with *p*-values below the Bonferroni significance level.

Compared with *Irf8^f/f^Itgax-Cre-R2^-/-^*control mice, the mortality of IRF8-targetted Cre+ mice was significantly improved (**Figure 1A**) and proteinuria were markedly decreased (**Figure 1B**). This result was consistent with an overall reduction of lethal kidney disease by conditional deletion of *Irf8*. We confirmed differences in kidney pathology by histological analyses and diagnosis encompassing several parameters, including endocapillary hypercellularity, karyorrhexis, fibrinoid necrosis, hyaline deposits, cellular/fibrocellular crescents, and interstitial inflammation. A combined pathologic score was assigned to each kidney. There was a greater reduction in pathological scores among mice with Cre-targeted *Irf8* deletion than in IRF8-sufficient controls (**Figures 1C, D**). The numbers of kidney-infiltrating immune cells including CD4T, CD8T, monocytes and cDC1s were also markedly reduced in *Irf8^f/f^Itgax-Cre+R2^-/-^* compared to controls (**Figure 1E**. Gating schemes were depicted in **Supplementary Figure S1**). Altogether, we concluded that Itgax-Cre-mediated IRF8 deletion abrogated lethal nephritis in R2^-/-^ mice.

**Figure 2:**
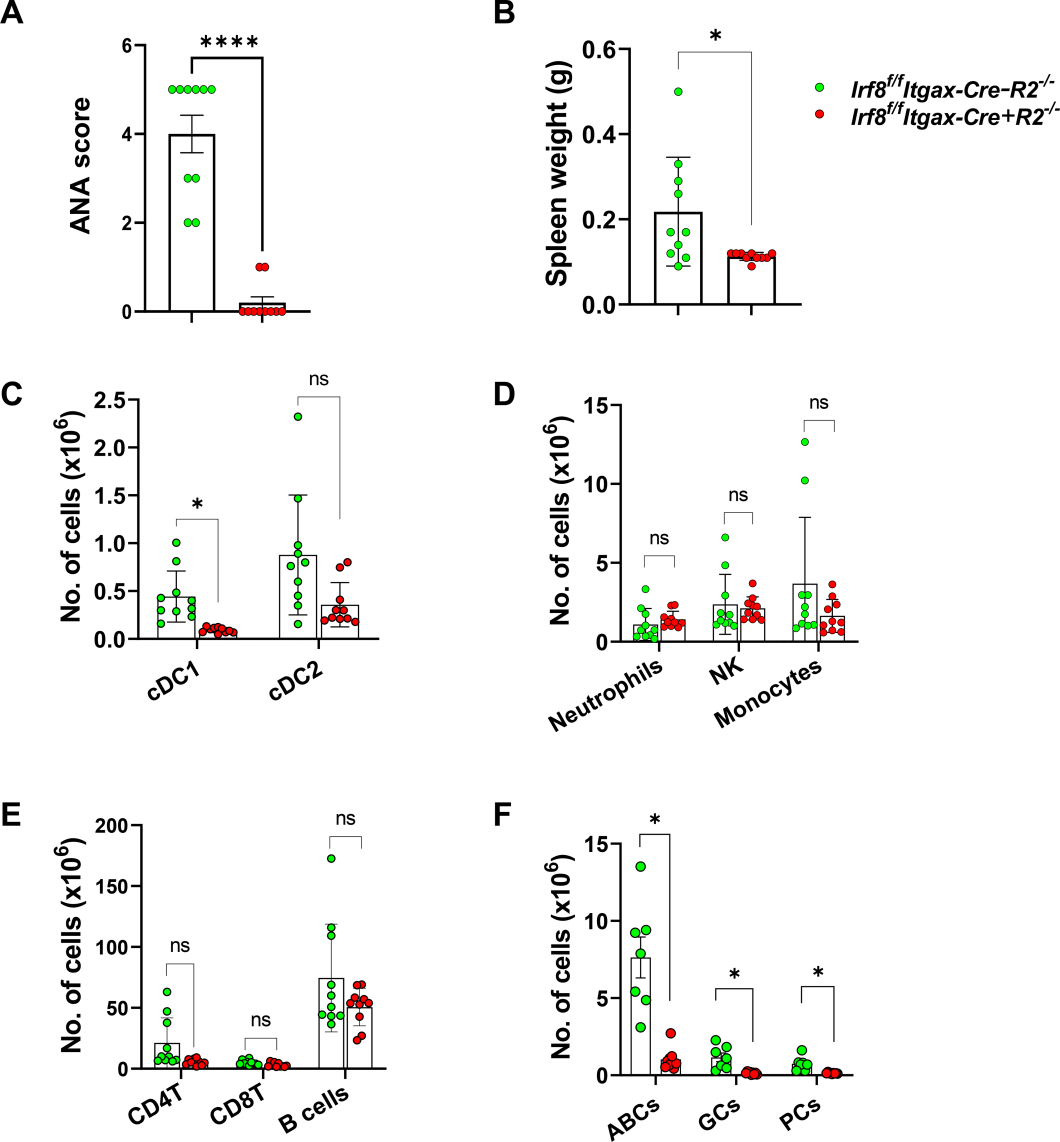
Reduced inflammatory response in IRF8-deficient mice. **(A)** The serum levels of ANA and **(B)** spleen weights were compared between *Irf8^f/f^Itgax-Cre+R2^-/-^* and control mice. **(C-F)** The cell numbers of defined immune cells in the spleen were compared between *Irf8^f/f^Itgax-Cre+R2^-/-^* and control mice. Each symbol represents a mouse. Statistical analysis was done by using unpaired *t* test in **(A, B)**, and **(C-F)** using multiple Mann-Whitney tests with Bonferroni’s correction. For **(A, B)**, *p < 0.05, ****p < 0.0001; for **(C-F)**, * denotes pairs with *p*-values below the Bonferroni significance level.

Since lupus pathology in R2^-/-^ mice is predated by the presence of autoantibodies in circulation, we determined the titers of serum ANAs. Indeed, Itgax-Cre targeted *Irf8* was correlated with almost complete abrogation of the phenotype (**Figure 2A**). Reduction in autoreactivity in Cre-expressing mice was also correlated with reduced splenomegaly (**Figure 2B**). Multiple-color flow cytometry analyses of splenocytes revealed several differences in immune cell numbers. Based on the expression of CD26, CD11b and CD24, cDCs (CD11c^+^MHC^+^) were subdivided into cDC1s (CD26^+^CD11b^-^CD24^+^) and cDC2s (CD26^+^CD11b^+^CD24^-^) (gating strategy was shown in **Supplementary Figure S1**). As expected, a near complete loss of cDC1s was found in *Irf8^f/f^Itgax-Cre+R2^-/-^* mice compared to controls (**Figure 2C**), consistent with the reported essential role for *Irf8* in cDC1 development (10). We found no significant differences in other immune cells in the spleen, such as cDC2s, monocytes and CD4Ts (**Figures 2D, E**; gating strategy shown in **Supplementary Figure S1**). While the total number of splenic B cells was similar in mice with conditional *Irf8* deletion compared to controls (**Figure 2E**), we decided to estimate the numbers of several activated B cell populations given the stark differences that we had observed in autoantibody titers. Atypical B cells (ABCs) express CD11c and have been associated with autoreactivity in other systems (22). They were almost eliminated by the Itgax-targeted deletion of *Irf8* in R2^-/-^ mice (**Figure 2F**). In addition, spontaneously activated B cells (germinal center (GC) and plasma cells) were markedly reduced in *Irf8^f/f^Itgax-Cre+R2^-/-^* mice compared to controls (Gating strategy was shown in **Supplementary Figure S1**) (**Figure 2F**). This result suggests that *Itgax*-Cre expression might result in *Irf8* deletion in B cells that normally don’t express CD11c.

Analyses of *Irf8^f/f^CD11c-Cre+R2^-/-^* mice revealed very similar results as *Irf8^f/f^Itgax-Cre+R2^-/-^* mice. *Irf8^f/f^CD11c-Cre+R2^-/-^* mice exhibited prolonged survival (**Supplementary Figure S2A**), significantly reduced proteinuria (**Supplementary Figure S2B**) and serum ANA levels (**Supplementary Figure S2C**). The spleen weights of *Irf8^f/f^CD11c-Cre+R2^-/-^* mice were significantly reduced compared with controls (**Supplementary Figure S2D**). Multiple comparisons among immune cells seemed to show a trend in reduction in CD4T, B cells, and monocytes between *Irf8^f/f^CD11c-Cre+R2^-/-^* and control mice (**Supplementary Figure S2E**). However, none of these comparisons reached the threshold for significant differences when the Bonferroni correction was applied. As expected, the frequencies of cDC1s were dramatically decreased, whereas those of cDC2s were slightly increased (**Supplementary Figure S2F**). The numbers of ABCs were also decreased (**Supplementary Figure S2H**). In the kidney of *Irf8^f/f^CD11c-Cre+R2^-/-^* mice, the numbers of infiltrated leukocytes and lymphocytes were significantly reduced in CD4T, CD8T, monocytes and cDC1s (**Supplementary Figure S2G**), consisting with the pathological presentations (**Supplementary Figure S2I, J**). Taken together, the autoimmune manifestations in both *Irf8^f/f^CD11c-Cre+R2^-/-^* and *Irf8^f/f^Itgax-Cre+R2^-/-^* mice were almost completely abrogated, highlighting the critical role of IRF8 in pathogenesis of lupus nephritis.

### Itgax-Cre-mediated deletion of *Irf8* occurs in many cell types including DCs, B cells and myeloid cells

**Figure 3:**
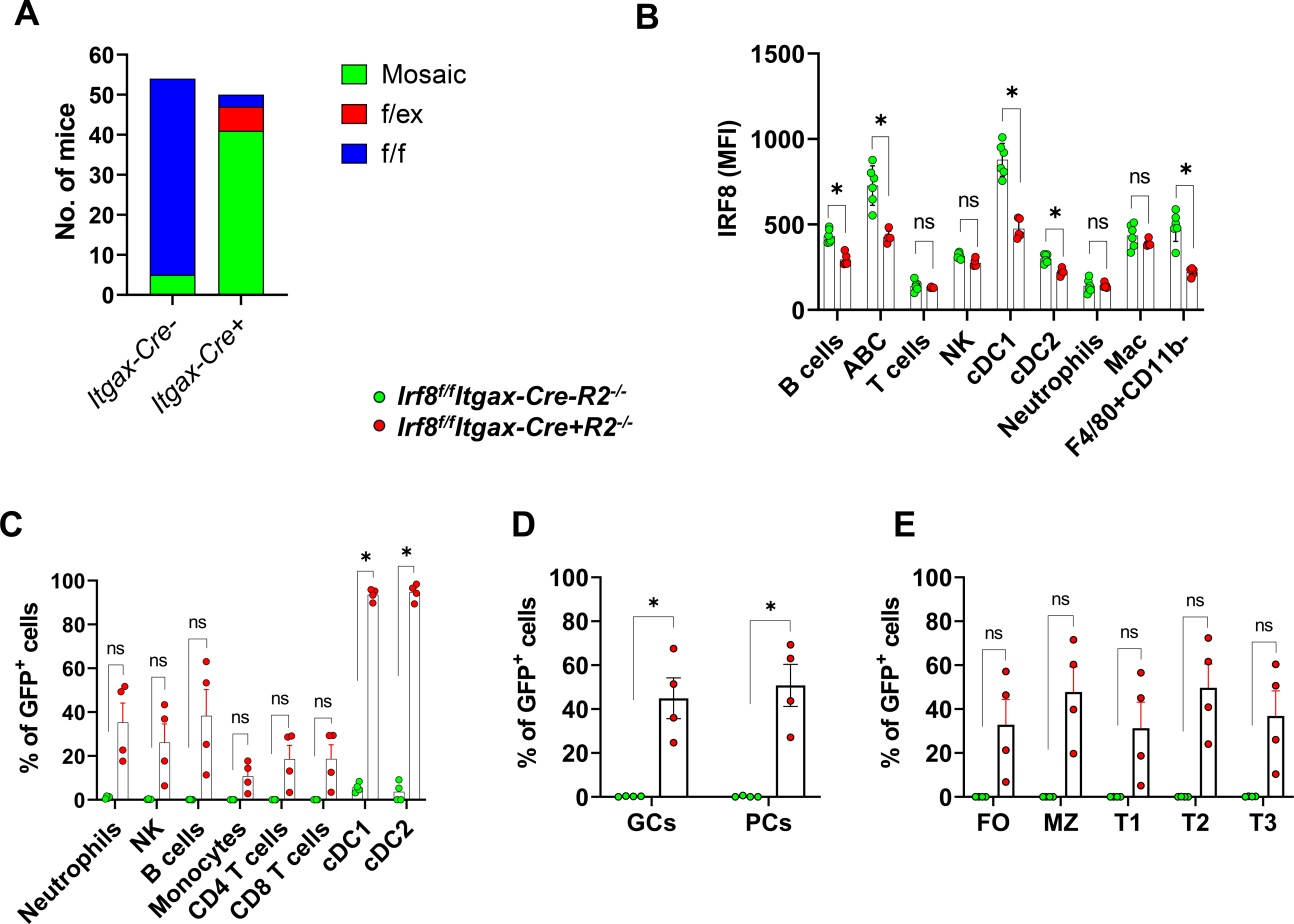
Expression of Itgax-Cre-EGFP and IRF8 in subpopulations of cells. **(A)** Detection of mosaic and excised *Irf8* alleles by PCR. The parents of these mice were all *Irf8^f/f^*. f, floxed allele; ex, excised allele. **(B)** Expression levels of IRF8 proteins were detected by intracellular staining and flow cytometry. MFI, mean fluorescence intensity. **(C–E)** Expression of Itgax-Cre-EGFP in subpopulations of immune cells were detected by flow cytometry. Gating schemes were shown in **Supplementary Figure S1** and described previously (38). GC (B220^+^GL7^+^CD95^+^), PC (CD138^+^B220^+/-^). Each dot represents a mouse. **(B–E)** Data are representative of more than three independent experiments. Statistical analysis was performed using multiple Mann-Whitney tests with Bonferroni’s correction. * denotes pairs with *p*-values below the Bonferroni significance level.

Analysis of tail genomic DNA revealed mixed genotype results in all *Irf8^f/f^Itgax-Cre+R2^-/-^* mice but not *Irf8^f/f^Itgax-Cre-R2^-/-^* controls, suggesting that mosaicism occurred when Itgax-Cre was present (**Figure 3A**). Flow cytometric analysis of IRF8 protein expression revealed a ~50% reduction of IRF8 in B cells, ABCs, cDC1s, cDC2s and F4/80^+^CD11b^-^ monocytes (**Figure 3B**). We confirmed mosaicism in EGFP expression of all immune populations: 100% in DCs, 30-50% in lymphocytes and 10-20% in monocytes (**Figure 3C**). We tested many B cell subpopulations as the main effect of *Irf8* deletion was observed in the production of autoantibodies (**Figure 2A**). All B cell developmental populations and activated fractions tested showed an average of 50% EGFP positive cells in mice that did not genotype as fully heterozygous (**Figures 3D, E**). These data suggested that Itgax-Cre was expressed in unidentified hematopoietic precursors that gave rise to progeny immune cells broader than the well-known CD11c^+^ DCs with a result of somatic mosaicism in all immune cells. This result was consistent with a previous report that the CD11c-Cre transgene was found to be expressed in a variety of cell types beyond the expected DCs (35).

### XCR1-Cre induces complete deletion of IRF8

To circumvent the “leakiness” issue of Itgax or CD11c-Cre, we crossed *Irf8^f/f^R2^-/-^* with *Xcr1-Cre+R2^-/-^* mice with the intention of deleting *Irf8* in the cDC1 lineage exclusively, as previously reported (30). However, after three generations, we unexpectedly found complete deletion of *Irf8* either at one copy or two copies among progeny mice (**Supplementary Figure S3**). This prevented production of meaningful mice for experiment.

### B cells from *Irf8^f/f^Itgax-Cre+* mice are impaired in producing ANA

**Figure 4:**
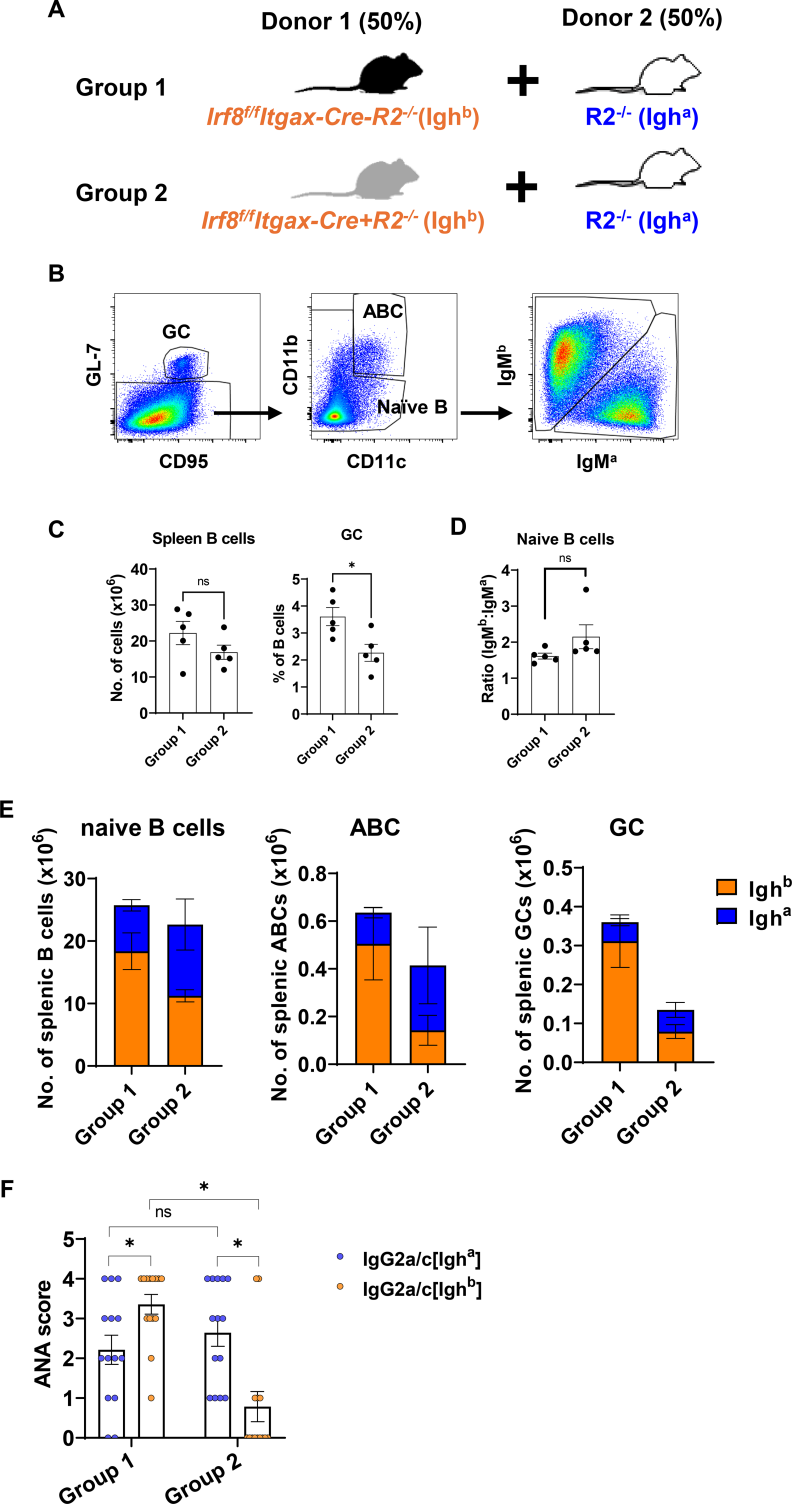
Inability of IRF8-deficient B cells to produce ANA *in vivo*. **(A)** Scheme of BM donors used to produce chimera mice. **(B)** Gating scheme used to define B cell subsets. All cells were gated on CD3^-^CD138^-^CD19^+^B220^+^ cells. **(C, E)** Numbers of splenic B cell subpopulations and **(D)** the ratio of IgM^b^/IgM^a^ naïve B cells of chimera mice were detected by flow cytometry. Data **(A-E)** are representative of three independent experiments. **(F)** Serum levels of ANA of each donor were detected by Hep2 cell staining and imaging analysis. Data are summary of three independent experiments. Each dot represents a recipient mouse. Statistical significance was calculated using unpaired *t* tests in **(C-E)** (*p < 0.05) and multiple Mann-Whitney tests with Bonferroni’s correction in **(F)** (* denotes pairs with *p*-values below the Bonferroni significance level). In panel **(E)**, *p* < 0.05 for Igh^b+^ ABCs and GCs, respectively, when comparing Group 1 versus Group 2.

To determine if IRF8 insufficiency and mosaicism in B cells affected autoantibody production, we generated chimeric mice using a 1:1 mixture of bone marrow cells from *Irf8^f/f^Itgax-Cre+R2^-/-^* (*Igh^b^* allotype), *Irf8^f/f^Itgax-Cre-R2^-/-^* (*Igh^b^* allotype), or wild-type R2^-/-^ (*Igh^a^* allotype) mice (**Figure 4A**). In this setting, B cell intrinsic effects due to Irf8 deficiency are observed as changes in the ratio between *Igh^a^* and *Igh^b^* allotypes among groups. These two alleles will produce distinct surface IgM allotypes in B cells measurable by flow cytometry. The two alleles also produce distinct released IgG antibodies measured by indirect immunofluorescence with anti-IgG_2a/c_^a^ and anti-IgG_2a/c_^b^ antibodies (lowercase script denotes antibody isotype, while superscript denotes gene allele and, consequently, the donor origin). Three months after reconstitution, splenocytes were analyzed by flow cytometry and serum ANA levels (IgG allotype “a” or “b”) were measured by staining Hep2 cells. The proportion of each donor allotype in various B cell populations was calculated with the gating scheme shown in **Figure 4B**. The yield of spleen B cells (**Figure 4C**) and the ratio of donors (IgM^b^: IgM^a^) in naïve B cells (**Figure 4D**) were comparable between Group1 and Group 2. However, the frequencies of GC B cells in Group 2 were reduced compared to Group 1 (**Figure 4C**). Although there was minimum skewing in naïve B cells, ABCs and GC cells were significantly reduced in chimeras that contained 50% cells originated from Itgax-Cre-expressing mice compared to those that contained control Cre-negative populations (**Figure 4E**). The reduction was exclusively observed in “b” allotypes (red colored data comparing group 1 and group 2) while “a” allotypes (colored blue) were mostly unchanged between group 1 and group 2. Furthermore, B cells from *Irf8^f/f^Itgax-Cre+R2^-/-^* donors produced significantly reduced levels of serum ANA antibodies (detected as IgG2a/c allotype “b” in Group 2) compared to IRF8-sufficent B cells (detected as IgG2a/c allotype “b” in Group 1) (**Figure 4F**). We did not observe B cell extrinsic effects of *Irf8* deletion in this experiment (i.e. effect of other cells that might express *Irf8*), because none of the phenotypes in the non-modified (both WT *Irf8*) allele “a” were reduced in group 2 compared to group 1. Taken together, these data suggest that IRF8 insufficiency in B cells impairs autoantibody production and that partial deletion of *Irf8* as mosaic expression is enough to fully abrogate spontaneous germinal centers and measurable autoreactivity.

## Discussion

In this study, we targeted cDC1s by using Cre-mediated IRF8 deletion systems to determine if cDC1s play a role in autoimmune glomerulonephritis. Consistent with previous research (10), we observed near 100% expression of Itgax-Cre-EGFP in total cDCs, resulting in a marked reduction of cDC1s in *Irf8^f/f^Itgax-Cre+R2^-/-^* mice. Remarkably, 30~50% of non-DCs, including B cells, also expressed Itgax-Cre-EGFP. Analyses of genomic DNA and EGFP expression driven by Cre revealed a likely mosaic deletion of *Irf8*, which was consistent with intracellular IRF8 protein levels. Interestingly, the reduction of IRF8 expression in half of the cells sufficiently blocked the development of lupus-like symptoms in *Irf8^f/f^Itgax-Cre+R2^-/-^* mice. The expansion of immune cells in the spleen and production of ANA were almost completely abolished. While the lack of autoimmune manifestations in *Irf8^f/f^Itgax-Cre+R2^-/-^* mice could be due to cDC1 deficiency, chimera mice using IRF8-sufficient and -deficient mixed bone marrow donors revealed an inability of the IRF8-deficient B cells to produce ANA *in vivo*. Therefore, the underdose of IRF8 in B cells clearly impaired autoantibody production, which could impair the development of lupus nephritis. Altogether, our results reemphasize the importance of understanding the “leaky” effect of Itgax- or CD11c-Cre on non-DC cells when using CD11c-Cre to target DC gene programs.

Whether cDC1s play a role in lupus nephritis is unclear. In a rheumatoid arthritis model, lack of cDC1s due to disrupted expression of Flt3 and Batf3 prevented collagen-induced arthritis (6). A similar result was reported in a primary biliary cholangitis model (7). Using anti-glomerular basement membrane antibodies, which induce acute glomerulonephritis, Chen et al. demonstrated that depletion of cDC1s attenuated renal inflammation (36). In our study, deletion of IRF8 using Itgax-Cre or CD11c-Cre abrogated glomerulonephritis (**Figure 1**; **Supplementary Figure S2**), which was also associated with both cDC1 and B cell deficiency (**Figures 1**, **4**). In mixed BM chimera mice, the presence of IRF8-sufficient cDC1s (Igh^a^) failed to facilitate ANA production by IRF8-deficient B cells (Igh^b^), implying two possible explanations. First, the mosaic deletion of *Irf8* in B cells may offset *Fc*γ*r2b* deficiency-conferred hyperactivity thereby preventing production of autoantibodies and eliciting glomerulonephritis. Second, a lack of cDC1s may fail to initiate autoreactive B cell differentiation into plasma cells. While the first possible explanation seems to be certain based on BM adoptive transfer experiments, the second explanation requires experiments to specifically deplete cDC1s without affecting B cells. Xcr1-Cre, which is thought to exclusively target cDC1s, unfortunately had similar “leaky” issues in deleting *Irf8* (**Supplementary Figure S3**).

CD11c-expressing ABCs are a unique population of B cells with a proposed association with pathogenesis of SLE (37). In addition to CD11c, ABCs also express CD11b and T-bet. Depletion of IRF5 or IRF8 using CD23-Cre profoundly reduced the number of ABCs and curbed autoimmune symptoms in DEF6/SWAP-70 double deficient mice (22). In our study, we observed a significant reduction of ABCs in *Irf8^f/f^Itgax-Cre+R2^-/-^* mice (**Figure 2F**) and *Irf8^f/f^CD11c-Cre+R2^-/-^* mice (**Supplementary Figure S2H**) compared to controls. BM chimera mice also showed poor development of IRF8-deficient ABCs (**Figure 4E**). These results suggest that IRF8 is required for the development of ABCs, consistent with previous report (22). However, it is currently unknown if the lack of ANA production in chimera mice, as well as naïve *Irf8^f/f^Itgax-Cre+R2^-/-^* mice, was due to an absence of ABCs or hampered B cell activation. Future studies are warranted to clarify this issue.

In summary, our results confirmed that IRF8 is required for B cells to produce autoantibodies. However, the role of cDC1s in the development of lupus-like disease in *R2^-/-^* mice is inconclusive due to the leakage of IRF8 deletion in B cells. The limitation of CD11c-Cre and Xcr1-Cre due to DC non-specific expression of CD11c and Xcr1 should be acknowledged when the strains are used to target DC genes.

## Data Availability

The raw data supporting the conclusions of this article will be made available by the authors, without undue reservation.
