# Supplementary material for: CD11c-Cre driven deletion of Irf8 reveals the effect of somatic mosaicism in a mouse model of SLE
Source: Front Immunol. 2026 Jan 26;16:1662894. doi: 10.3389/fimmu.2025.1662894 (PMC12883780; doi:10.3389/fimmu.2025.1662894)
Supplement: Supplementary file 1 [file DataSheet1.pdf]

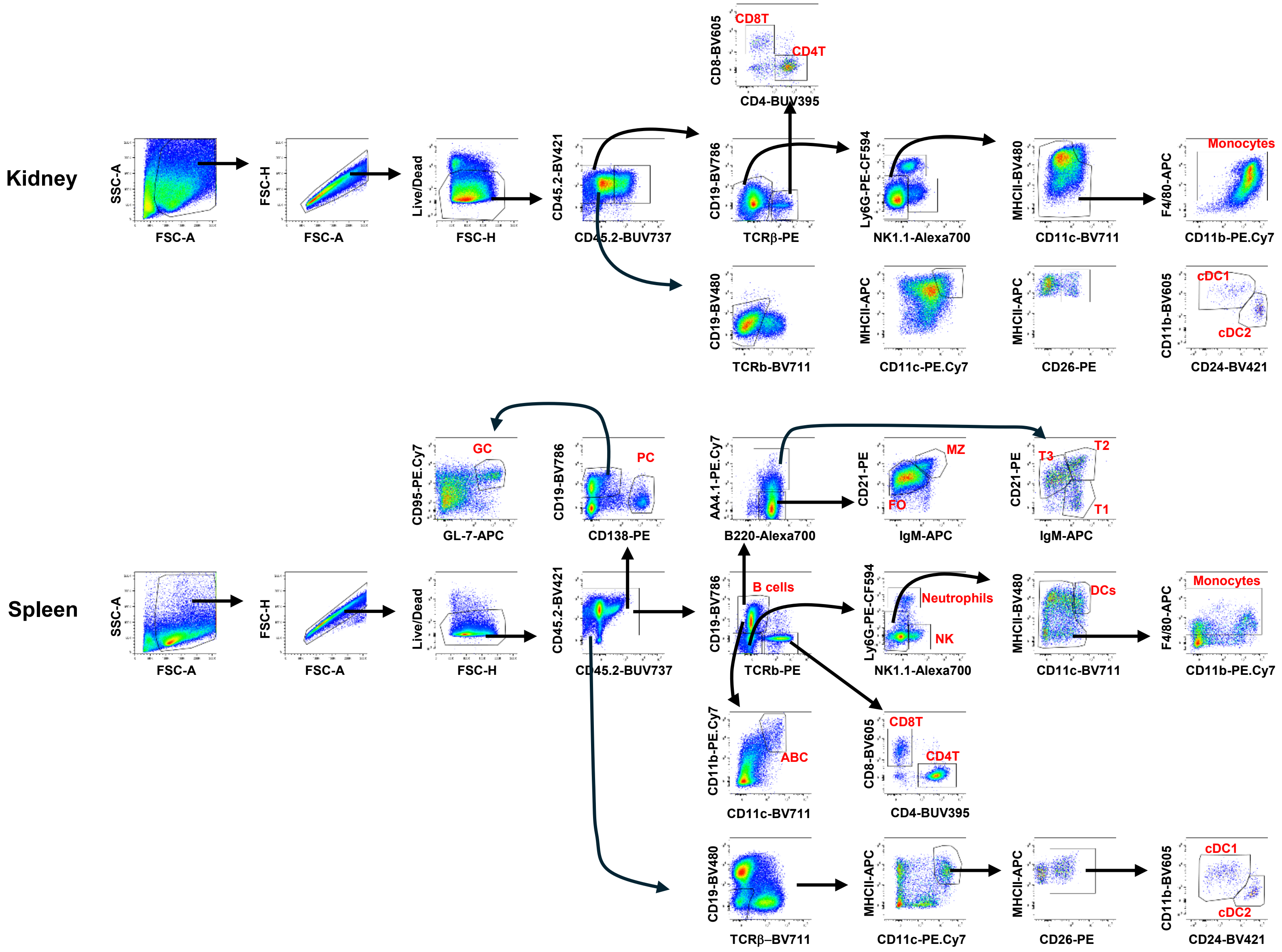

Supplemental Fig. S1. Gating schemes of spleen and kidney cells. Serial gating was performed to exclude dead cells/debris and doublets. Kidney resident cells/infiltrated cells were gated on CD45-BUV733 negative cells. For spleen cells, the CD45-BUV733 signals were ignored.

Suppl Fig S2

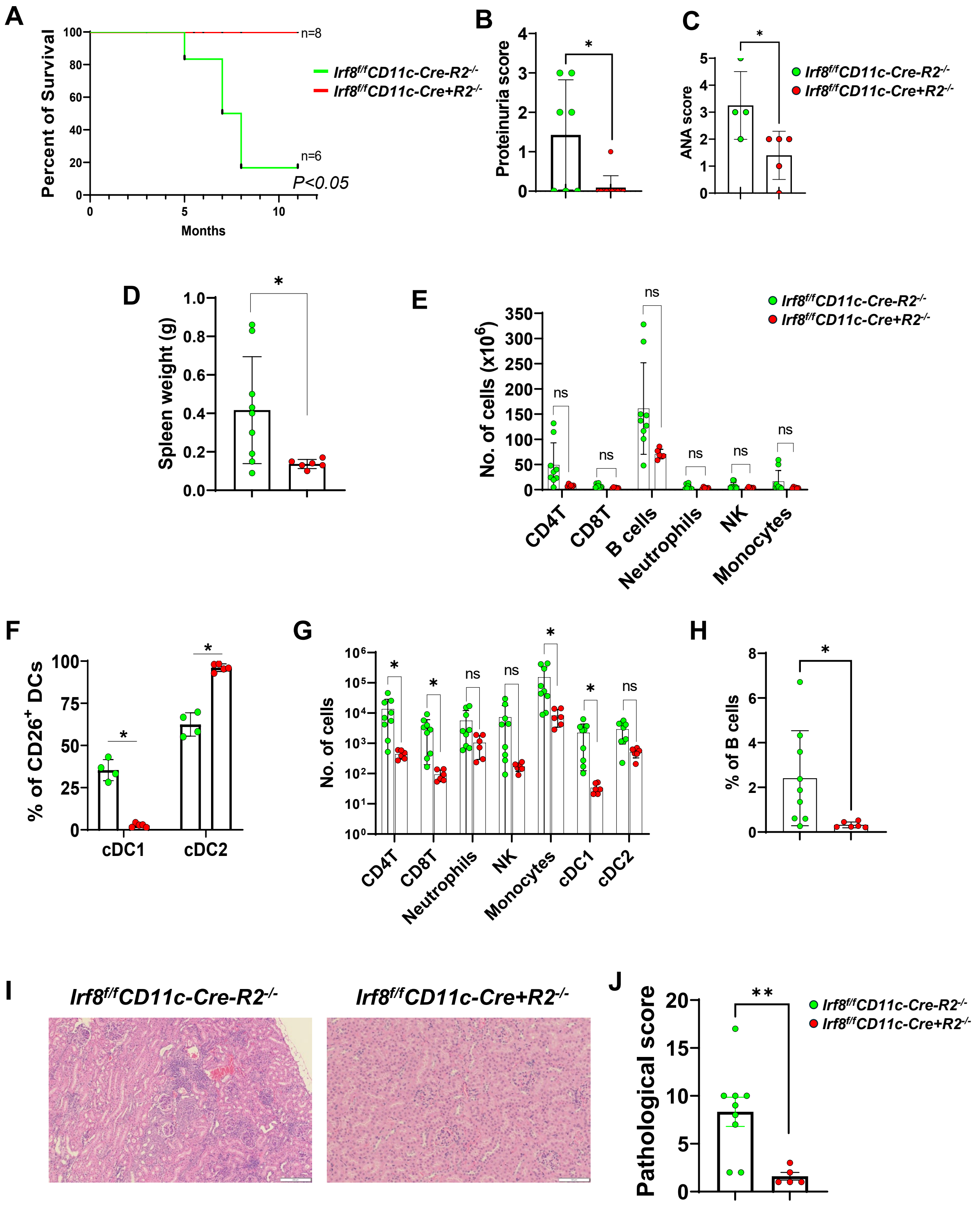
A
Percent of Survival
Months
n=8
n=6
P<0.05
Irf8f/fCD11c-Cre-R2-/-
Irf8f/fCD11c-Cre+R2-/-
B
Proteinuria score
*
C
ANA score
*
D
Spleen weight (g)
*
E
No. of cells (x10⁶)
ns
CD4T
CD8T
B cells
Neutrophils
NK
Monocytes
F
% of CD26+ DCs
cDC1
cDC2
G
No. of cells
cDC1
cDC2
H
% of B cells
I
Irf8f/fCD11c-Cre-R2-/-
Irf8f/fCD11c-Cre+R2-/-
J
Pathological score
**

Supplemental Fig. S2. Autoimmunity is abrogated by IRF8 deficiency. (A) Survival curves of *Irf8*$^{f/f}$*CD11c-Cre+R2*$^{-/-}$ mice were compared to control mice. $p<0.05$. (B) Proteinuria scores and (C) the serum levels of ANA were significantly decreased in *Irf8*$^{f/f}$*CD11c-Cre+R2*$^{-/-}$ mice. (D) Spleen weights of *Irf8*$^{f/f}$*CD11c-Cre+R2*$^{-/-}$ mice were significantly lower than control mice. (E) The cell numbers of each immune cell population in the spleen were determined by flow cytometry. (F) The absolute numbers of cDC subsets in the spleen of *Irf8*$^{f/f}$*CD11c-Cre+R2*$^{-/-}$ and control mice. (G) Numbers of infiltrated subpopulations of cells in each kidney were detected by flow cytometry. (H) Detection of ABCs by flow cytometry. (I) Representative H&E-stained sections of kidneys in *Irf8*$^{f/f}$*CD11c-Cre-R2*$^{-/-}$ and *Irf8*$^{f/f}$*CD11c-Cre+R2*$^{-/-}$ mice. (H) NIH activity index values of each kidney samples studied. Each symbol represents a mouse (B-H, J). Unpaired student *t* test (B, C, D, F, H, J) and multiple Mann-Whitney test (E and G) with Bonferroni correction tests were performed to show statistical differences. For B, C, D, H and J, *$p<0.05$, **$p<0.01$,***$p<0.001$. For E, F and G, * denotes pairs with *p*-value below the Bonferroni significance level.

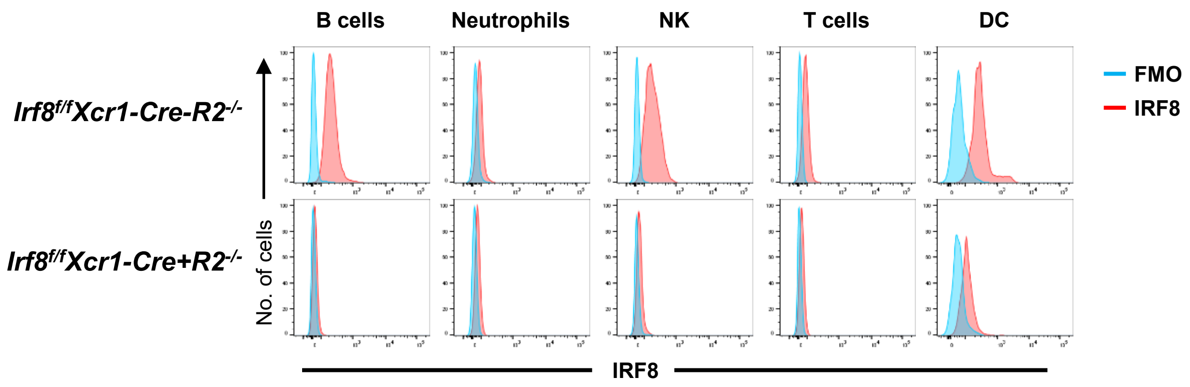

Supplemental Fig. S3. Deletion of IRF8 in *Irf8$^{f/f}$Xcr1-Cre+R2$^{-/-}$* mice. IRF8 was detected by intracellular staining and flow cytometry. FMO, fluorescence minus one control. Data represents three or more mice of two independent experiments.
